# Wafer-Scale Integration of Inverted Nanopyramid Arrays for Advanced Light Trapping in Crystalline Silicon Thin Film Solar Cells

**DOI:** 10.1186/s11671-016-1397-6

**Published:** 2016-04-12

**Authors:** Suqiong Zhou, Zhenhai Yang, Pingqi Gao, Xiaofeng Li, Xi Yang, Dan Wang, Jian He, Zhiqin Ying, Jichun Ye

**Affiliations:** Key Laboratory for Microstructures, Institute of Materials, Shanghai University, Shanghai, 200072 China; Ningbo Institute of Material Technology and Engineering, Chinese Academy of Sciences, Ningbo, 315201 People’s Republic of China; College of Physics, Optoelectronics and Energy & Collaborative Innovation Center of Suzhou Nano Science and Technology, Soochow University, Suzhou, 215006 China; Key Lab of Advanced Optical Manufacturing Technologies of Jiangsu Province & Key Lab of Modern Optical Technologies of Education Ministry of China, Soochow University, Suzhou, 215006 China

**Keywords:** Light trapping, Crystalline silicon thin film, Solar cells, Inverted nanopyramids, Nanosphere lithography

## Abstract

Crystalline silicon thin film (c-Si TF) solar cells with an active layer thickness of a few micrometers may provide a viable pathway for further sustainable development of photovoltaic technology, because of its potentials in cost reduction and high efficiency. However, the performance of such cells is largely constrained by the deteriorated light absorption of the ultrathin photoactive material. Here, we report an efficient light-trapping strategy in c-Si TFs (~20 μm in thickness) that utilizes two-dimensional (2D) arrays of inverted nanopyramid (INP) as surface texturing. Three types of INP arrays with typical periodicities of 300, 670, and 1400 nm, either on front, rear, or both surfaces of the c-Si TFs, are fabricated by scalable colloidal lithography and anisotropic wet etch technique. With the extra aid of antireflection coating, the sufficient optical absorption of 20-μm-thick c-Si with a double-sided 1400-nm INP arrays yields a photocurrent density of 39.86 mA/cm^2^, which is about 76 % higher than the flat counterpart (22.63 mA/cm^2^) and is only 3 % lower than the value of Lambertian limit (41.10 mA/cm^2^). The novel surface texturing scheme with 2D INP arrays has the advantages of excellent antireflection and light-trapping capabilities, an inherent low parasitic surface area, a negligible surface damage, and a good compatibility for subsequent process steps, making it a good alternative for high-performance c-Si TF solar cells.

## Background

In order to maintain the rapid development of photovoltaic (PV) market in a sustainable way, the reduction of silicon used in solar cells is one of the key recent concerns in PV communities. The latest studies suggest that crystalline silicon (c-Si) thin film (TF) solar cells with an active thickness of a few micrometers may provide a viable pathway to promote the cost reduction and maintain high efficiencies [1–4]. In addition to remarkably save the material, thinner wafers have many added advantages, e.g., efficient carrier diffusion and collection can be realized under a short transport length, allowing the utilization of low-quality materials for PV products (i.e., an important factor determining the cell cost); the crystalline silicon thin film (c-Si TF) solar cells are light in weight with a high flexibility, suitable for applications in military, aerospace, and other special circumstances.

However, with decreasing the wafer thickness, the absorption of the Si cells to sunlight is dramatically decreased (especially in the long-wavelength band) due to the indirect-band property of c-Si [5]. This strongly invokes the advanced light-trapping schemes in order to substantially strengthen the light absorption of c-Si TFs. The state-of-the-art technique for the conventional bulk c-Si wafers is the surface texturing, which produces randomly sized (3–10 μm) pyramids and can obtain an extremely low device reflection in broadband. However, these large-size pyramids are obviously unsuitable for c-Si TF solar cells, because of the unacceptable material waste, increased fragment probability, etc. Two-dimensional (2D) nanophotonic crystals have recently opened unprecedented opportunities for boosting the light absorption, notably in c-Si TFs [6–9]. A variety of nanostructure designs, such as nanopillars [10], nanowires [11, 12], nanocones [2, 13, 14], nanoholes [4, 15–18], nanodomes [19], nanopyramids [20–24], and nanopencils [25, 26], have been used at the front and/or back side of the absorber layer to refract, diffract, and reflect light, with the aim of increasing the total optical path length within the cell [27–29].

Optical optimizations on high-performance c-Si TF solar cells have revealed that inverted nanopyramid (INP) arrays could be a good choice for surface texturing due to their progressive profile that provided the best combination of antireflection and light-trapping properties [20, 30, 31]. In addition, the INP arrays show the advantages in inherently low parasitic surface areas, negligible surface damages, and good compatibility with subsequent processing steps, which minimized electronic losses and simplified fabrication procedures [20, 21, 32]. Furthermore, with the latest development of high-throughput large-area technique of colloidal lithography in our lab [33], INP arrays could be readily fabricated at manufacturing scale to meet the requirement on high throughput in photovoltaic industry.

In this letter, wafer-scale INP arrays with three typical periodicities (300, 670, and 1400 nm) were fabricated on front, rear, or both surfaces of c-Si thin films with a thickness of ~20 μm. Systematic design and evaluation on optical properties of c-Si TFs with different INP arrays were performed via both full-wave finite-element method (FEM) simulation and experiments. It was revealed that the periodicity of 1400 nm delivers the highest light absorption, under a good agreement between simulation and experiment. With incorporating an antireflection coating (ARC) layer onto the top surface, the 20-μm-thick c-Si TF doubly textured with INP arrays (1400 nm in periodicity) yielded an absorption spectrum approaches (exceeds) the Lambertian limit in the wavelength range of 400 ≤ λ ≤ 900 nm (λ > 900 nm). The corresponding photocurrent density (*J*_ph_) predicted from the absorption spectrum is as high as 39.86 mA/cm^2^, which is about 75 % higher than that of the flat counterpart (22.63 mA/cm^2^).

## Methods

### c-Si Thin Film Fabrication

The ultrathin c-Si films were thinned from double-side polished p-type (100) Si wafers (4 in. diameter, 200-μm thickness, 1–5 Ω cm resistivity, CZ) with a chemical etching (KOH solution with a concentration of 50 wt% at 80 °C). The etching rate is about 80 μm/h. The etching process was performed in a well-designed setup in our lab; the resultant thin wafer has a high uniformity in thickness because of the well-controlled temperature distribution in the whole solution, with the help of a continuous stirring. The thickness of these thin films was judged by the transmittance color of wafer through white light.

### Polystyrene Nanosphere Monolayer Fabrication

**Fig. 1:**
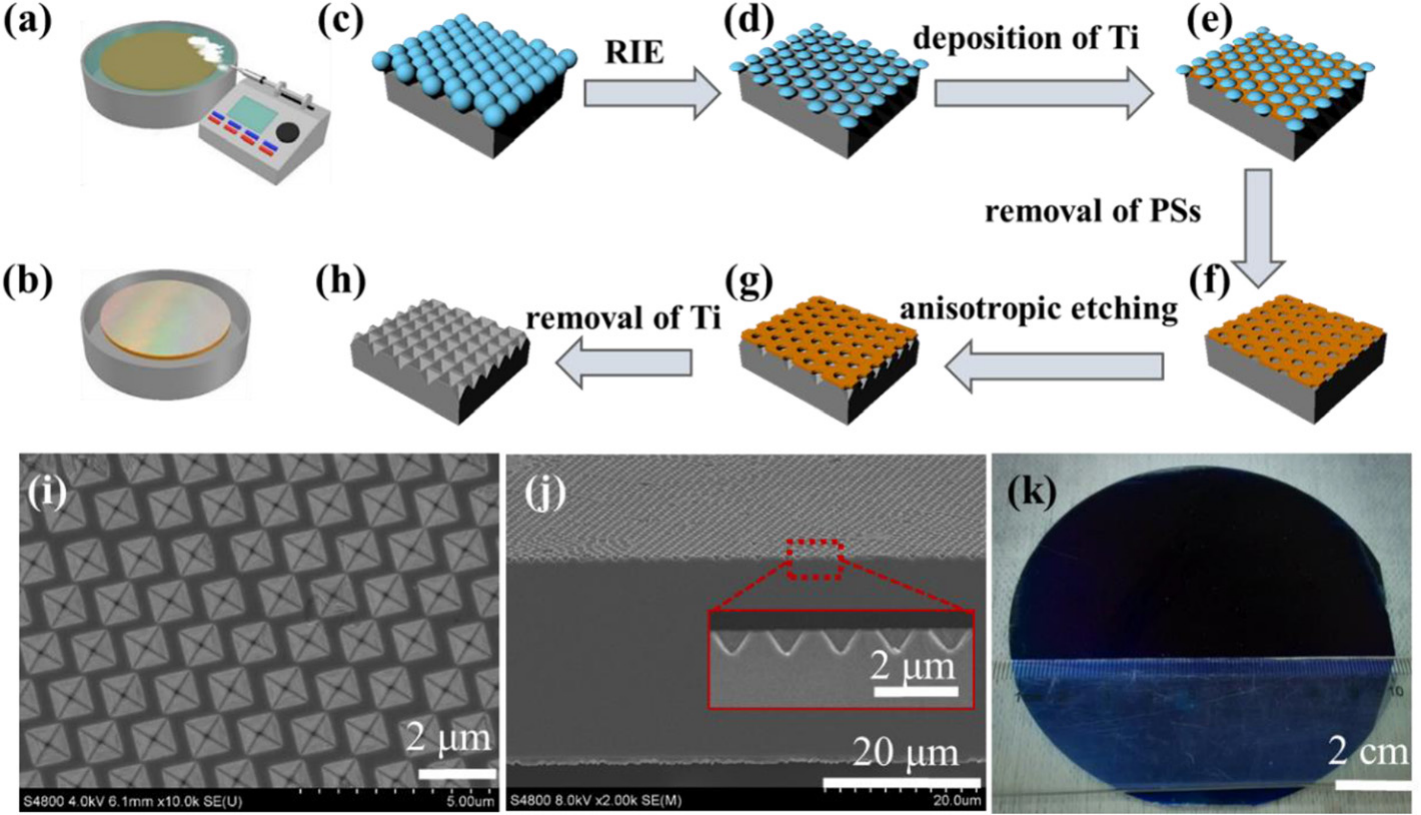
Schematics of the fabrication processes for PS nanosphere arrays (**a**–**b**) and the fabrication of INP arrays (**c**–**h**). Top-viewed (**i**) and cross-sectional (**j**) SEM image for hexagonal INP arrays with periodicity of 1400 nm and optical image (with ARC) of 20-μm-thick c-Si textured with INP arrays (**k**). The *scale bar* is 2 μm in **i**, 20 μm in **j**, and 2 cm in **k**

A large-area monolayer of hexagonally close-packed polystyrene (PS) nanosphere arrays was fabricated via a Micro-propulsive Injection technique that were developed in our lab [33]. Briefly, as shown in Fig. 1a, b, the well-mixed aqueous suspension of polystyrene nanospheres (2.5 wt%, synthesized by dispersion polymerization) and alcohol (water *vs* alcohol, 1:1 in volume) was directly injected on the water surface, and then, PS nanospheres were self-arranged into close-packed hexagonal arrays at the air/water interface. The large-area monolayer can be transferred onto the preset Si wafers (supported by a glass slide) by slowly declining the water level or raising the substrate.

### Inverted Nanopyramid (INP) Array Fabrication

Figure 1c–h gives a schematic illustration of the fabrication flow of inverted nanopyramid (INP) arrays. The size of the PS nanospheres in the hexagonally close-packed arrays was reduced to have the gaps between the nanospheres by a plasma etching in the atmosphere mixed of O_2_/Ar (process d). A 50-nm titanium was then deposited by magnetron sputtering, followed by an ultrasonic wash in methylbenzene to remove the PS spheres (process e and f). The Si thin films with meshy titanium mask were then anisotropically etched in a 20 wt% NaOH: 20 wt% IPA solution at 60 °C for 6~10 min. Since the etch rate in the (100) direction is many times higher than in the (111) direction, INPs were formed eventually (process g). To fabricate the doubled-sided INP structure, when one side of the ultrathin c-Si film was deposited with meshy titanium mask (process f), the sample was turn to the other side and repeated the process (c–f) and the front and rear surfaces with titanium masks were then anisotropic wet etched into INP arrays simultaneously (process g). Finally, the nanotextured thin film Si wafers were immersed in 10 % HF solution for 10 min to remove the titanium mask (process h).

The silicon nitride layer on the front surface and silicon dioxide layer on the rear surface of samples was deposited by PECVD. Conformal Ag layer (back reflector) was deposited by magnetron sputtering.

### Measurements

The optical characterization of the samples was carried out using spectrophotometer (Helios LAB-rc, with an integrating sphere) in the 375- to 1100-nm wavelength range. Because all of the samples measured here have a Ag metal layer on the back as the back reflector to prevent light transmission, the absorptance (A) of the final structure can be simply calculated from the *A* = 1 − *R*, where *R* is the reflectance. The morphologies of the samples were observed using a Field Emission Scanning Electron Microscope (FE-SEM, Hitachi S-4800).

### Simulation Method

In numerical simulations, the optical performance is predicted by using the full-wave finite-element method (FEM) in COMSOL Multiphysics [34]. The spectral response of c-Si solar cells ranges from 375 to 1100 nm, corresponding to the bandgap (1.12 eV) of the photoactive material. Meanwhile, the wavelength-dependent refractive indices of all materials are from the Palik’s data [35]. To reproduce the experimental structures, we always perform three-dimensional (3D) simulation in this study. In the model, a unit cell of the nanostructure is built and applied with two pairs of periodic boundary conditions (PBC) to mimic the periodic nature in different lattice directions. Perfectly matched layer (PML) together with scattering boundary condition (SBC) are employed to avoid the unphysical reflection at the front and rear edges of the computational domain, respectively. Furthermore, for the normally incident light, only one polarization needs to be considered under such a symmetric device configuration. To evaluate the overall performance, the *J*_ph_ is calculated by integrating the simulated absorption spectrum of the cell with standard AM 1.5G illumination [36] under the assumption of a perfect internal carrier-transport process.

## Results and Discussion

### Morphology of the Inverted Nanopyramid Arrays

Figure 1a–h gives a schematic illustration of the fabrication flow of inverted nanopyramid (INP) arrays. A hexagonally close-packed monolayer of polystyrene (PS) nanospheres was used as a lithographic mask for the anisotropic wet etching (in NaOH) to fabricate periodic INP arrays on the surfaces of 20-μm-thick c-Si films. Typical PS nanospheres with three different sizes were selected to achieve INP arrays with periodicities of 300 (sub-wavelength), 670 (mid-wavelength), and 1400 nm (infrared-wavelength), respectively, in order to study the periodicity-related light-trapping properties. Figure 1a, b shows the fabrication of PS monolayer via a Micro-propulsive Injection technique that we developed previously, with the possibility of assembling PS nanospheres in both large scale and high throughput [33]. Figure 1c–h shows the patterning process, which will be described in details in the “Methods” section. The typically resultant INP arrays with the periodicity of 1400 nm are demonstrated in Fig. 1i, j with a top-view and a cross-sectional scanning electron microscope (SEM) images, respectively. The INP arrays are shown to be well arranged in a hexagonal manner, stemmed from the PS nanosphere masks. The time of NaOH wet etching has to be well controlled until INPs merged in the direction of one diagonal, as clearly shown in Fig. 1i, f, in order to achieve the maximum-sized but intact INPs. As a result, the fill factor of the INP on the c-Si surface was limited to 0.7. Note that the cross-sectional SEM image in Fig. 1j is along the direction of the side edges of INP arrays. The interlinked property of lateral dimension and depth related to NaOH wet etching causes an angle of 54.7° to the wafer surface for each INP, and the facets are terminated with the silicon (111) surface. Figure 1h displays an optical image of a 20-μm-thick c-Si film (4-in.) textured with INP arrays (*P* = 1400 nm) and covered by an antireflection coating (ARC) layer (80 nm thick SiN_x_). The wafer exhibits a uniform dark blue under a low device reflection. The demonstration of 4-in. wafer shows the potential of this fabrication process to be used in large-area manufacturing environments.

### Antireflection Property on the Front Surface

**Fig. 2:**
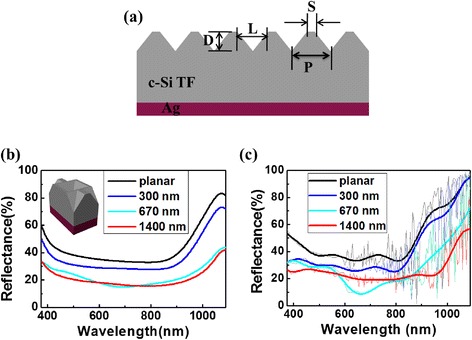
Schematic representation of the periodic INP arrays with back reflector (**a**), showing all the critical dimensions used in the simulation. Comparison of the reflection spectra of structured 20-μm-thick c-Si thin films with front-sided INP arrays with periodicity of 300, 670, and 1400 nm, respectively, for experiments (**b**) and simulations (**c**)

Firstly, the effect of periodic INP arrays on the front surface antireflection property was characterized in both simulations and experiments, without including ARC. The critical dimensions used in the simulation, including the periodicity (*P*), depth (*D*), side length (*L*), and the separation between side edges in two adjacent INPs (*S*) were denoted in Fig. 2a with *L* ~ 0.7*P* and *S* = *P* – *L*. In Fig. 2b (experiment) and Fig. 2c (simulation), the reflection spectra of the INP arrays with *P* = 300, 670, and 1400 nm were compared, respectively. The experimental curves show excellent agreement with the theoretical predictions over almost the entire spectrum ranging from 375 to 1100 nm. The small deviations are most likely caused by the fluctuations of the separation between INPs. All the 20-μm-thick c-Si films textured with different INP arrays show certain reflection reduction over the planar counterpart, which can be explained by the roughened surfaces and the gradual change of the refractive index from air to Si. This gradual transition of refractive index, also known as impedance matching, leads to a better light coupling inside the c-Si TFs [28]. For the INP arrays with *P* = 300 nm, the feature size is too small to effectively couple with light, and thus, the incident light can easily penetrate through the film and then reflect back under the function of Ag back reflector layer, resulting in only a marginal absorption enhancement. As *P* increases to 670 nm, light absorption is improved over the entire solar spectrum, because the feature size is more close to the mid-wavelength (which corresponds to the strongest energy of sunlight spectrum), introducing a better light coupling inside the active layer. Particularly, a reflection valley is observed at the range of 600 ≤ *λ* ≤ 700 nm, both from experimental and simulated curves, which is about equal to the periodicity (670 nm), indicating that an enhanced light scattering occurs when *λ*~*P*. The minimum overall reflection of 20.29 % (42.32 % for planar) is observed when *P* = 1400 nm, benefited from the more prominent gradient effective refractive index profile between air and silicon due to the deeper INPs.

### Light-Trapping Property on the Rear Surface

**Fig. 3:**
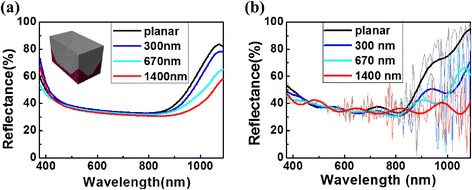
Experimental (**a**) and simulated (**b**) reflection spectra of 20-μm-thick c-Si thin films textured by INP arrays with different periodicities on the rear side. The *inset* in **a** shows a unit for the periodic array of c-Si and rear-sided INP (coated by a conformal silver film)

As the c-Si has low absorption coefficient, especially in the near-infrared region, c-Si TF cells rely crucially on advanced light management scheme to achieve high conversion efficiencies. Therefore, another set of INP arrays is applied onto the rear side of c-Si TF to enhance the diffractions for the light with *λ* > 800 nm. Similarly, three periodicities of *P* = 300, 670, and 1400 nm were formed on the rear side [see the inset of Fig. 3a with a silver nanostructured reflector]. The corresponding absorption spectra of experiment and simulation are plotted in Fig. 3a, b, respectively. As predicted, the light reflection for the band of *λ* < 800 nm exhibits unnoticeable change with the presence of rear INP design, as light in this band has been efficiently absorbed by the photoactive layer before reaching the bottom facet. However, for wavelengths ranging from 800 to 1100 nm, distinct absorption enhancements over the planar counterpart are observed, especially for the design with *P* = 1400 nm. It is expected that light scattering is improved since the pyramidal shape represents a gradual change from the uniform Si base to the periodic INP grating at the apex, rather than an abrupt change in planar systems. This allows the normally incident light to be coupled and guided laterally, resulting in an increased effective optical path. The oscillation of the simulated absorption spectra in Fig. 3b further confirmed the strong interference of light throughout the INP arrays.

### Light Harvesting of Double-Sided Inverted Nanopyramid Arrays

**Fig. 4:**
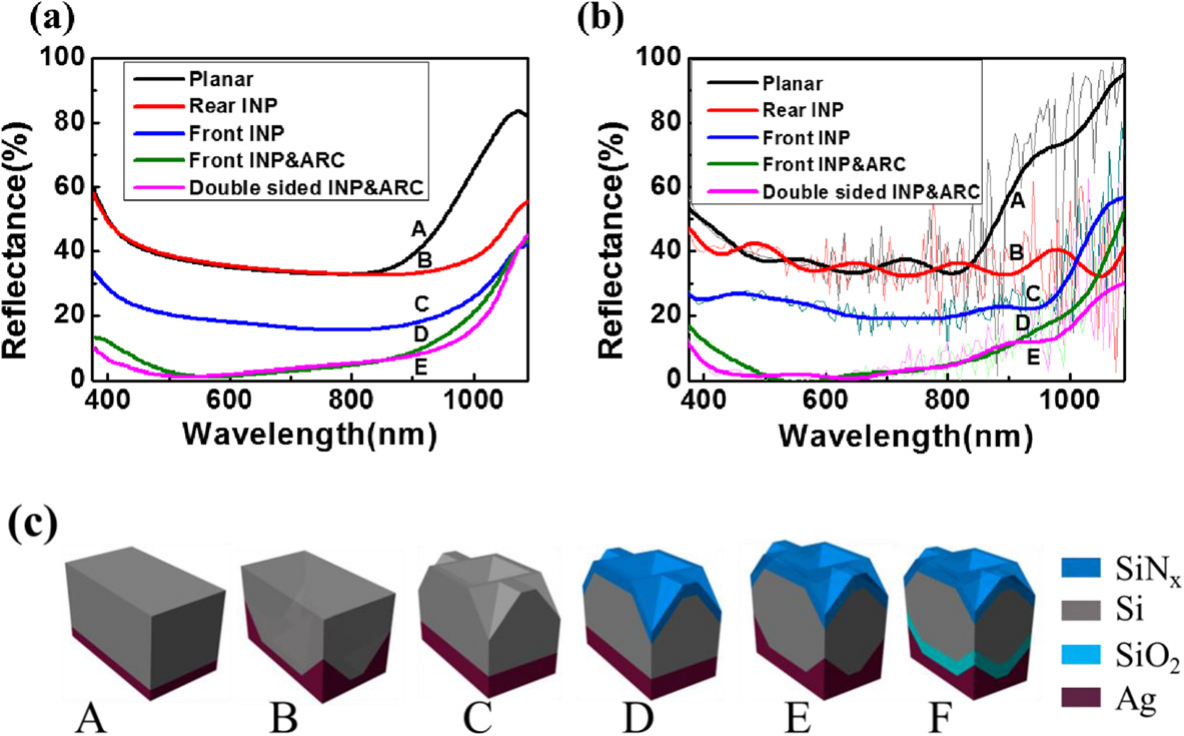
Experimental (**a**) and simulated (**b**) reflection spectra of 20-μm-thick c-Si thin films with different surface texturing structures (*A*–*E*) that are schematically denoted in **c. c** The schematic units for different light-trapping designs, with planar film and rear Ag reflector (*A*), planar film and rear INP and Ag reflector (*B*), rear INP and rear Ag reflector (*C*), front INP with SiNx antireflector and rear Ag reflector (*D*), front INP with SiNx antireflector and rear INP with Ag reflector (*E*), front INP with SiNx antireflector and rear INP with SiO_2_ separator and Ag reflector (*F*). Structure F is separated addressed in Fig. 6. The periodicity of INP array is 1400 nm for both sides

According to the aforementioned results, the optimum periodicities for INP arrays configured on the front and rear surfaces are both 1400 nm. To achieve the absorption enhancement over the whole band of c-Si, a double-sided texturing based on INP arrays with *P* = 1400 nm were thus fabricated on 20-μm-thick c-Si TFs, where the front nanostructure served for antireflection in the entire spectrum and the rear one for long-wavelength light trapping. For summarization, the light absorption properties of five samples shown in Fig. 4c, i.e., 20-μm-thick planar c-Si TF (A), textured by rear-sided INP (B), front-sided INP (C), front-sided INP and ARC (D), and double-sided INP and ARC (E), were plotted in Fig. 4a (experiment) and Fig. 4b (simulation). Again, consistent results have been achieved from both experiment and FEM simulation. It is clear that structure D shows an outstanding absorption enhancement over structure C, due to additional antireflection effect from an 80-nm ARC layer of SiN_x_. Structure E consistently outperforms the structures A–D on reflection over the entire band. Because of the existence of ARC, the difference between structures E and D in visible band is negligible. However, when *λ* ≤ 500 nm or 900 ≤ λ ≤ 1100 nm, structure E exhibits a lower reflection over structure D. The long-wavelength performance enhancement is ascribed to the light-trapping effect of rear INP arrays; nevertheless, the short-wavelength enhancement could be associated with the interference enhancement between transmitted light from the front surface and the reflected light from the rear surface, leading to the increased internal reflections.

**Fig. 5:**
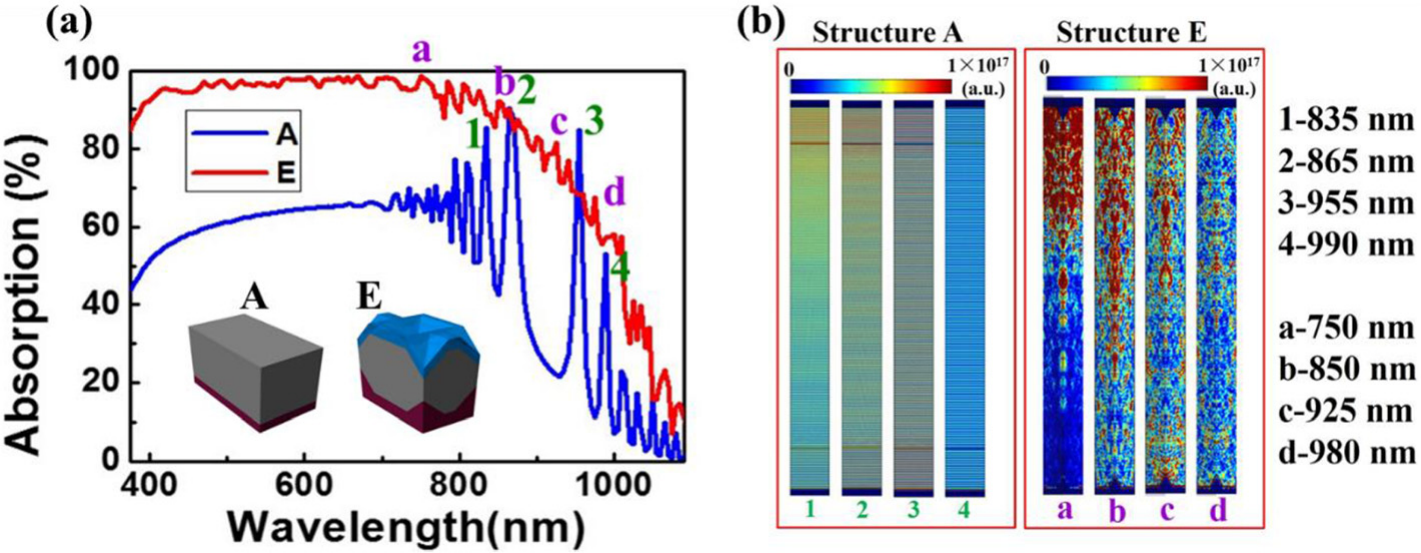
**a** Comparison of the absorption spectra of structures A and E. **b** Optical absorption profiles inside the structure A (*left*) and the structure E (*right*), respectively. The wavelength selected for structure A is of 1—835 nm, 2—865 nm, 3—955 nm, and 4—990 nm, while for structure E is of a—750 nm, b—850, c—925 nm, and d—980 nm

To further illustrate the propagation nature of c-Si TF with double-sided INPs and ARC, the simulated absorption spectra and spatial profiles of structures A and E are compared in Fig. 5a, b, respectively. It can be clearly seen from Fig. 5a that light absorption has been dramatically improved with the double-INP design, with only one exception at *λ* ~ 955 nm where a strong Fabry-Perot (F-P) cavity resonance has been formed in the planar system. The absorption spatial profiles at four representative wavelengths (i.e., *λ* = 835, 865, 955, and 990 nm for structure A, and *λ* = 750, 850, 925, and 980 nm for structure E) are shown in Fig. 5b. For structure A, the incident photons penetrate deeper into the c-Si layer, which leads to typical F-P resonances in planar system. The F-P resonances can only induce few absorption peaks at typical wavelengths where the normal incident light strongly interferences with the reflected light by the rear flat surface. However, structure E (c-Si layer with double-sided INP arrays and ARC) behaves in a different way. The absorption efficiency of structure E is much improved, and the absorption pattern is more irregular compared to structure A (planar c-Si), indicating that structure E is composed of much richer interfaces and cavity modes rather than the standard planar multilayer cavity. As shown in the right portion of Fig. 5b, for *λ* = 750 nm, a careful examination on the penetration depth shows that front INP arrays elongate the light-path and improve the absorption, while the contributions from the rear INP arrays is negligible. Differently, INP-like patterns are observed in almost the whole photoactive region for the absorption profiles at *λ* = 850, 925, and 980 nm, and such pattern becomes more obvious on the rear side with increasing wavelength, indicating the enhanced scattering to long-wavelength light by the INP arrays.

### Characterizations of the Optically Parasitic Loss

**Fig. 6:**
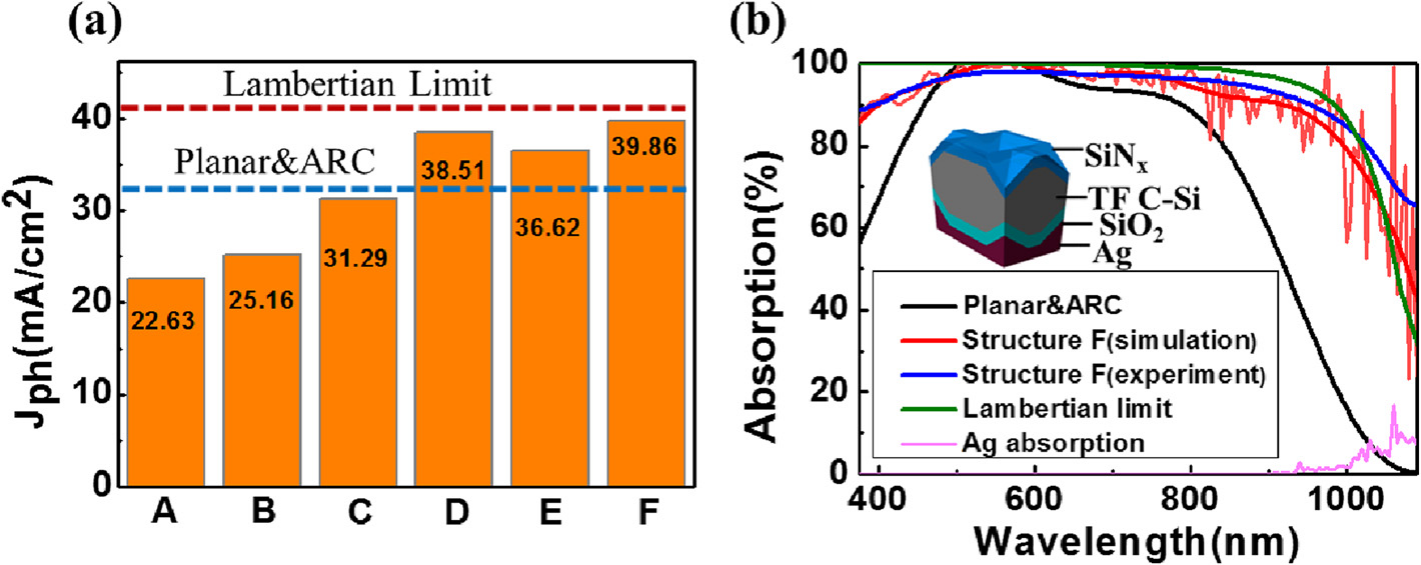
**a** Comparison of the photocurrent density theoretically generated for the six structures denoted in Fig. 4 (*A*—planar; *B*—rear INP; *C*—front INP; *D*—front INP and SiN_x_; *E*—double-sided INP and SiN_x_; *F*—double-sided INP and SiN_x_ andSiO_2_), the Lambertian limit (*red line*) and the planar and ARC structure (*blue line*) are also shown. **b** Comparison of the simulated and experimental absorptance spectra of structure F, the absorptance spectra of planar and ARC structure and the Lambertian limit for 20-μm-thick c-Si are also shown

The *J*_ph_ generated from different structures was calculated from the light absorption based on the finite-element method (FEM). The *J*_ph_ of the six structures (as denoted in Fig. 4c) was compared in Fig. 6a and, the details were summarized in Table 1. To make the comparison of structures combined with ARC layer fairer, additional sample of structure A together ARC (planar and ARC) was selected as the reference line (blue dashed-line in Fig. 6a). The *J*_ph_ value for the planar system (structure A) is only 22.63 mA/cm^2^, with the major loss (i.e., 23.20 mA/cm^2^) from the severe surface reflection. As to structure B (C), *J*_ph_ increases to 25.16 (31.29) mA/cm^2^, assisted by the light-trapping (antireflection and guiding) effect of the rear (front) INP arrays. With an extra contribution of ARC (Structure D), *J*_ph_ further improved to 38.51 mA/cm^2^. It is worth pointing out that the *J*_ph_ loss at the rear side increases from 0.57 (planar) to 4.00 (structure B) and 0.29 (planar and ARC) to 5.27 mA/cm^2^ (structure E), due to the increased parasitic absorption in the conformal Ag nanostructure under plasmonic resonances [37]. As a result, structure E yields a moderate *J*_ph_ of 36.62 mA/cm^2^ that is even lower than structure D, despite it has the lowest reflection [see Fig. 4b]. In order to minimize the parasitic absorption at the rear side of structure E, a SiO_2_ layer (1 μm) was used to separate the rear INP and Ag layer, as demonstrated by structure F in Fig. 4c. With the help of the spacing layer of SiO_2_, structure F finally yields a *J*_ph_ of 39.86 mA/cm^2^, which is 23.8 % higher than that of the planar and ARC system and is only 3 % below the Lambertian limit (41.1 mA/cm^2^ for 20-μm-thick c-Si) [38]. For comparison, more clearly, we selected the absorption spectra of structure F, planar and ARC, and Lambertian limit and displayed them in Fig. 6b. It is seen that, under the improved light-trapping performance as well as the negligible parasitic absorption in Ag, structure F exhibits very similar (superior) absorption to the Lambertian system when 500 nm < λ < 800 nm (*λ* > 1000 nm). The slightly lower *J*_ph_ from structure F is the relatively lower absorption in the sub-wavelength and near-infrared wavelength region, i.e., *λ* < 500 nm and 800 nm < *λ* < 1000 nm.

**Table 1:** The comparison on simulated photocurrent density gains and losses for the seven surface texturing

*J*_ph_(mA/cm^2^)	Planar and ARC	A	B	C	D	E	F
*J*_ph_ (Si)	32.19	22.63	25.16	31.29	38.51	36.62	39.86
*J*_ph_ (top loss)	1.92	23.20	17.17	13.10	5.61	4.23	5.57
*J*_ph_ (rear loss)	0.29	0.57	4.00	1.89	2.20	5.27	0.89

## Conclusions

In conclusion, we investigated both experimentally and theoretically the light-harvesting properties of 20-μm-thick c-Si thin films textured with periodic inverted nanopyramid (INP) arrays fabricated by a colloidal lithography and anisotropic wet etching technique. The periodicities of the INP arrays on front and rear surface were separately optimized for light absorption enhancement in broadband and red/near-infrared regions, respectively. With the incorporation of the antireflection coating, the c-Si thin films textured with optimal double-sided INP arrays obtained nearly perfect light absorption that is very close to the Lambertian limit among most of the wavelength range and even beyond the Lambertian limit at long-wavelength band. Combined with the other attributes of less material damage, inherently low parasitic surface area, scalable fabrication capability, and good compatibility with subsequent process steps, the double-sided 2D nanophotonic crystals of inverted nanopyramids is believed to be a promising way to texture c-Si thin film solar cells with high energy-conversion efficiency and high potential in cost reduction.

## Abbreviations

ARC: antireflection coating
c-Si TF: crystalline silicon thin film
FEM: full-wave finite-element method
FE-SEM: field emission scanning electron microscope
F-P: Fabry-Perot
INP: inverted nanopyramid
*J*_ph_: photocurrent density
PBC: periodic boundary conditions
PML: perfectly matched layer
PS: polystyrene
PV: photovoltaic
SBC: scattering boundary condition

## References

[1] Wang S, Weil BD, Li Y, Wang KX, Garnett E, Fan S (2013). Large-area free-standing ultrathin single-crystal silicon as processable materials. Nano Lett.

[2] Jeong S, McGehee MD, Cui Y (2013). All-back-contact ultra-thin silicon nanocone solar cells with 13.7 % power conversion efficiency. Nat. Commun.

[3] Catchpole K, Polman A (2008). Plasmonic solar cells. Opt. Express..

[4] Chen T-G, Yu P, Chen S-W, Chang F-Y, Huang B-Y, Cheng Y-C (2014). Characteristics of large-scale nanohole arrays for thin-silicon photovoltaics. Prog. Photovolt: Res. Appl..

[5] Priolo F, Gregorkiewicz T, Galli M, Krauss TF (2014). Silicon nanostructures for photonics and photovoltaics. Nat Nanotechnol.

[6] Narasimhan VK, Cui Y (2013). Nanostructures for photon management in solar cells. Nanophotonics.

[7] Brongersma ML, Cui Y, Fan S (2014). Light management for photovoltaics using high-index nanostructures. Nat Mater.

[8] Han SE, Chen G (2010). Toward the Lambertian limit of light trapping in thin nanostructured silicon solar cells. Nano lett..

[9] Jeong S, Wang S, Cui Y (2012). Nanoscale photon management in silicon solar cells. J. Vac. Sci. Technol. A..

[10] Shir D, Yoon J, Chanda D, Ryu JH, Rogers JA (2010). Performance of ultrathin silicon solar microcells with nanostructures of relief formed by soft imprint lithography for broad band absorption enhancement. Nano lett..

[11] Kelzenberg MD, Boettcher SW, Petykiewicz JA, Turner-Evans DB, Putnam MC, Warren EL (2010). Enhanced absorption and carrier collection in Si wire arrays for photovoltaic applications. Nat. Mater..

[12] Garnett E, Yang P (2010). Light trapping in silicon nanowire solar cells. Nano lett..

[13] Wang KX, Yu Z, Liu V, Cui Y, Fan S (2012). Absorption enhancement in ultrathin crystalline silicon solar cells with antireflection and light-trapping nanocone gratings. Nano Lett.

[14] Wang B, Leu PW (2012). Enhanced absorption in silicon nanocone arrays for photovoltaics. Nanotechnology..

[15] Trompoukis C, El Daif O, Depauw V, Gordon I, Poortmans J (2012). Photonic assisted light trapping integrated in ultrathin crystalline silicon solar cells by nanoimprint lithography. Appl. Phys. Lett..

[16] Hong L, Wang X, Zheng H, Wang J, Wang H, Yu H (2014). Optical absorption enhancement in a Si nanohole structure with hexagonal unit cell for solar cell application. Nanotechnology..

[17] Han SE, Chen G (2010). Optical absorption enhancement in silicon nanohole arrays for solar photovoltaics. Nano lett.

[18] Yahaya NA, Yamada N, Kotaki Y, Nakayama T (2013). Characterization of light absorption in thin-film silicon with periodic nanohole arrays. Opt Express.

[19] Zhu J, Hsu CM, Yu Z, Fan S, Cui Y (2010). Nanodome solar cells with efficient light management and self-cleaning. Nano lett.

[20] Mavrokefalos A, Han SE, Yerci S, Branham MS, Chen G (2012). Efficient light trapping in inverted nanopyramid thin crystalline silicon membranes for solar cell applications. Nano lett..

[21] Branham MS, Hsu WC, Yerci S, Loomis J, Boriskina SV, Hoard BR (2015). 15.7 % efficient 10-mum-thick crystalline silicon solar cells using periodic nanostructures. Adv Mater.

[22] Branham MS, Hsu W-C, Yerci S, Loomis J, Boriskina SV, Hoard BR (2016). Empirical comparison of random and periodic surface light-trapping structures for ultrathin silicon photovoltaics. Adv. Optical. Mater..

[23] Cariou R, Chen W, Cosme-Bolanos I, Maurice J-L, Foldyna M, Depauw V (2016). Ultrathin PECVD epitaxial Si solar cells on glass via low-temperature transfer process. Prog. Photovolt: Res. Appl..

[24] Weinstein LA, Hsu WC, Yerci S, Boriskina SV, Chen G (2015). Enhanced absorption of thin-film photovoltaic cells using an optical cavity. J. Opt..

[25] Lin H, Xiu F, Fang M, Yip S, Cheung HY, Wang F (2014). Rational design of inverted nanopencil arrays for cost-effective, broadband, and omnidirectional light harvesting. ACS Nano.

[26] Lin H, Cheung H-Y, Xiu F, Wang F, Yip S, Han N (2013). Developing controllable anisotropic wet etching to achieve silicon nanorods, nanopencils and nanocones for efficient photon trapping. J. Mater. Chem. A..

[27] Li X, Hylton NP, Giannini V, Lee K-H, Ekins-Daukes NJ, Maier SA (2011). Bridging electromagnetic and carrier transport calculations for three-dimensional modelling of plasmonic solar cells. Opt Express.

[28] Li X, Hylton NP, Giannini V, Lee K-H, Ekins-Daukes NJ, Maier SA (2013). Multi-dimensional modeling of solar cells with electromagnetic and carrier transport calculations. Prog. Photovolt: Res Appl.

[29] Shang A, Zhai X, Zhang C, Zhan Y, Wu S, Li X (2015). Nanowire and nanohole silicon solar cells: a thorough optoelectronic evaluation. Prog. Photovolt: Res. Appl..

[30] Li G, Li H, Ho JY, Wong M, Kwok HS (2014). Nanopyramid structure for ultrathin c-Si tandem solar cells. Nano lett.

[31] Wang P, Azimi S, Breese MB, Peters M (2014) Theoretical investigation of “nano-muffin” and inverted nano-pyramid surface textures for energy harvesting in very thin c-Si solar cells. Materials Research Society Proceedings, vol 1638, Cambridge Univ Press. doi:10.1557/opl.2014.244

[32] Trompoukis C, El Daif O, Pratim Sharma P, Sivaramakrishnan Radhakrishnan H, Debucquoy M, Depauw V (2015). Passivation of photonic nanostructures for crystalline silicon solar cells. Prog. Photovolt: Res. Appl..

[33] Gao P, He J, Zhou S, Yang X, Li S, Sheng J (2015). Large-area nanosphere self-assembly by a micro-propulsive injection method for high throughput periodic surface nanotexturing. Nano lett..

[34] COMSOL Multiphysics. http://www.comsol.com/. Accessed 8 Apr 2016

[35] Palik ED (1985) Handbook of optical constants of solids. Academic Press, Orlando

[36] ASTM, Reference solar spectral irradiance: AM 1.5 Spectra.http://rredc.nrel.gov/solar/spectra/am1.5. Accessed 8 Apr 2016

[37] Shang A, Li X (2015). Carrier depletion and electrical optimization of gallium arsenide plasmonic solar cell with a rear metallic grating. Appl. Phys. Lett..

[38] Wang L, Han J, Lochtefeld A, Gerger A, Carroll M, Stryker D, Bengtson S, Curtin M, Li H, Yao Y (2013). 16.8% efficient ultra-thin silicon solar cells on steel 28th EU PVSEC, 3DV 1.

